# Enhancing Cognitive Impairment Assessment and Management in Hip Fracture Patients: A Two-Cycle Clinical Audit

**DOI:** 10.7759/cureus.95316

**Published:** 2025-10-24

**Authors:** Saif Abdulsattar, Utkarsh Shahi, Shahid Mir

**Affiliations:** 1 Trauma and Orthopaedics, Manchester University NHS Foundation Trust, Manchester, GBR

**Keywords:** amts, audit, cognitive assessment, delirium, hip fracture, quality improvement

## Abstract

Background: Cognitive impairment significantly affects elderly hip fracture patients, dramatically worsening their outcomes, yet healthcare teams consistently fail to screen for it. Despite national guidelines mandating routine cognitive assessment, compliance remains alarmingly low. This audit confronted this critical gap between evidence and practice in fragility hip fracture care.

Methods: We conducted a prospective two-cycle audit at Northwick Park Hospital in Harrow, England, to transform cognitive assessment practices. Cycle 1 (July-November 2023) exposed baseline failures in 30 patients aged ≥65 with fragility hip fractures. With these insights, we deployed a multifaceted intervention: electronic alerts that could not be ignored, intensive staff training, and strategic visual reminders throughout the unit. Cycle 2 (November 2023-January 2024) tested whether these changes could continue in 25 similar patients. We tracked Abbreviated Mental Test Score (AMTS) documentation at four critical junctures: admission, postoperative days 1 and 2, and the day 4 orthogeriatric review.

Results: The change occurred in a noticeable way, but it was not brought to completion. Initial AMTS documentation improved from 14 (46.7%) to 18 (72%) between cycles. Most dramatically, postoperative day 1 assessments, completely absent at baseline, reached nine (36%) completions, while day 2 assessments climbed from zero to four (16%). However, orthogeriatric day 4 assessments actually declined from 19 (63.3%) to 13 (52%), suggesting intervention fatigue. The shift to electronic records proved double-edged-boosting admission assessments while paradoxically disrupting established day 4 documentation routines.

Conclusions: Strategic interventions can revolutionize cognitive assessment practices, particularly for initial screening, but sustaining these gains demands more than one-time fixes. While education and visual cues successfully embedded new behaviors, the persistent gaps in postoperative reassessment reveal the challenge of truly transforming clinical culture. Success requires not just implementing change but hardwiring it through workflow integration, electronic health record (EHR) optimization, and relentless performance monitoring.

## Introduction

Hip fractures represent one of the most devastating injuries affecting older adults worldwide, with profound implications for morbidity, mortality, and healthcare resource utilization. As the global population ages, hip fractures have emerged as a major public health challenge, with incidence rates projected to increase substantially over the coming decades. Beyond the immediate orthopedic trauma, hip fractures precipitate a complex cascade of medical complications that fundamentally alter patients' functional status, independence, and survival prospects. 

Evidence base for the systematic cognitive assessment 

Recognition of the critical importance of cognitive function in hip fracture outcomes has led to the development of comprehensive clinical guidelines emphasizing systematic cognitive assessment. The British Orthopaedic Association Standards for Trauma (BOAST) Guidelines for Hip Fracture Standards explicitly recommend cognitive screening as a fundamental component of comprehensive geriatric assessment [1]. Similarly, the National Institute for Health and Care Excellence (NICE) has published detailed guidance on hip fracture management and delirium prevention, highlighting the central role of cognitive assessment in optimizing patient outcomes [2,3]. 

The Abbreviated Mental Test Score (AMTS), developed by Hodkinson, remains the most widely utilized cognitive screening tool in UK clinical practice [4]. This validated 10-point assessment offers several practical advantages for busy clinical environments: rapid administration (typically two to five minutes), minimal training requirements, and established reliability for detecting cognitive impairment in elderly patients. The British Geriatrics Society has endorsed the AMTS as the preferred cognitive screening tool for acute care settings, with scores of 6 or below indicating significant cognitive dysfunction requiring further evaluation and intervention [5]. 

Substantial evidence demonstrates that systematic cognitive screening, when coupled with appropriate preventive interventions, can significantly reduce delirium incidence and associated complications. Multicomponent delirium prevention programs have achieved 30-50% reductions in delirium rates, with corresponding improvements in functional outcomes and healthcare resource utilization. The Hospital Elder Life Program represents the most extensively studied intervention model, demonstrating sustained effectiveness across diverse healthcare settings [6]. 

Clinical burden of hip fracture 

Hip fracture is a major cause of morbidity and mortality in older adults, with one-year mortality rates approaching 20-30% and fewer than half of survivors regaining their pre-fracture level of mobility and independence [7]. The immediate clinical challenges extend far beyond orthopedic management, encompassing complex medical comorbidities, perioperative complications, and prolonged rehabilitation requirements. Contemporary evidence emphasizes that optimal outcomes depend not only on surgical expertise but also on comprehensive geriatric assessment and multidisciplinary care coordination. 

The vulnerability of hip fracture patients is amplified by their advanced age, multiple comorbidities, and the physiological stress imposed by major trauma and surgery. This population faces heightened risks of cardiovascular complications, pulmonary embolism, surgical site infections, and functional decline. However, among the most significant yet potentially preventable complications is cognitive impairment, which affects a substantial proportion of patients and serves as a critical determinant of clinical outcomes. 

Cognitive impairment in hip fracture patients 

Cognitive impairment, including dementia, mild cognitive impairment, and delirium, affects up to 40% of hip fracture patients and represents one of the most important modifiable risk factors for adverse outcomes [8,9]. This high prevalence reflects the convergence of multiple risk factors: advanced age, pre-existing cognitive vulnerability, the stress of acute illness and hospitalization, surgical trauma, anesthesia exposure, pain, and medication effects. 

Delirium, the most common and potentially preventable form of acute cognitive impairment, develops in 13-50% of hip fracture patients and is associated with a two- to threefold increase in hospital mortality [10]. The clinical significance of cognitive impairment in this population cannot be overstated. Patients with cognitive dysfunction experience substantially increased risks of post-operative complications, prolonged hospital stays, higher mortality rates, and reduced likelihood of returning to independent living [11]. 

Implementation challenges in clinical practice 

Despite compelling evidence and clear professional guidance, implementation of systematic cognitive assessment in hip fracture care remains inconsistent and suboptimal. International studies reveal substantial variation in cognitive screening practices, with completion rates ranging from 25% to 75% across different healthcare systems [12]. This implementation gap represents a critical quality chasm that potentially denies thousands of patients access to evidence-based preventive interventions. 

Multiple barriers contribute to this implementation challenge. Organizational factors include competing clinical priorities, time constraints, insufficient staffing levels, and inadequate integration of cognitive assessment into routine clinical workflows. Individual-level barriers encompass knowledge gaps regarding assessment techniques, uncertainty about score interpretation and appropriate follow-up actions, and concerns about the perceived burden of additional documentation requirements [13]. 

Electronic health record (EHR) systems represent both an opportunity and a challenge for cognitive assessment implementation. While modern systems offer potential for automated prompting, standardized documentation, and clinical decision support, many current implementations lack user-friendly cognitive assessment tools and fail to provide meaningful guidance for clinical action based on assessment results [13,14]. 

Local context and performance gap 

At Northwick Park Hospital's Orthogeriatric Unit, preliminary audit data revealed significant deficiencies in cognitive assessment practice that aligned with broader national trends. Initial AMTS completion rates were approximately 47%, falling substantially below the 80% benchmark recommended by professional guidelines. More concerning was the virtual absence of systematic post-operative cognitive reassessment, with completion rates near zero despite evidence highlighting the critical importance of early delirium detection in the immediate post-surgical period. 

Theoretical framework for quality improvement 

Successful implementation of systematic cognitive assessment requires a comprehensive understanding of the multilevel factors influencing clinical behavior change. Quality improvement science emphasizes that sustainable practice change typically requires interventions addressing multiple barriers simultaneously rather than focusing on isolated factors. EHR optimization offers particular promise for overcoming common implementation barriers by providing point-of-care prompting, standardizing assessment protocols, and facilitating consistent documentation practices [14]. 

The combination of technological solutions with traditional educational and feedback approaches represents a theoretically sound strategy for driving comprehensive practice improvement. Electronic alerts can address the "forgetting" component of non-compliance, while staff education targets knowledge and confidence deficits. Audit and feedback mechanisms leverage professional accountability and peer comparison to motivate sustained engagement with quality improvement efforts. 

International perspectives and regulatory context 

The importance of cognitive assessment in hip fracture care has gained increasing recognition in national quality monitoring frameworks. The National Hip Fracture Database (NHFD) includes cognitive assessment completion as a core quality indicator, while regulatory bodies increasingly view systematic cognitive screening as an essential component of safe, high-quality care [7]. International comparisons reveal that healthcare systems with mandatory cognitive assessment reporting and financial incentives linked to compliance achieve substantially higher performance levels. 

The economic argument for systematic cognitive assessment is compelling and multifaceted. Cognitive complications significantly increase healthcare costs through prolonged hospitalization, increased nursing requirements, additional investigations, and higher readmission rates. Economic modeling studies suggest that effective delirium prevention programs can achieve cost savings of £1,200-2,500 per patient through reduced length of stay and complication rates [15]. 

Innovation opportunities and technological solutions 

Contemporary healthcare delivery increasingly emphasizes the integration of clinical decision support tools and quality improvement technologies into routine practice workflows. EHR systems offer unprecedented opportunities for systematic quality enhancement through automated prompting, standardized assessment protocols, and real-time performance feedback [14,16]. However, successful technology implementation requires careful attention to clinical workflow integration, user experience design, and ongoing system optimization based on frontline feedback. 

Research evidence increasingly supports multifaceted intervention approaches that combine technological solutions with traditional quality improvement methods. Studies examining EHR interventions alone typically achieve modest improvements (10-20% absolute increase), while comprehensive programs incorporating multiple intervention components can achieve substantially greater gains [17,18]. 

Patient safety and quality implications 

The patient safety implications of inadequate cognitive assessment are profound and far-reaching. Unrecognized cognitive impairment increases risks of medication errors, falls, treatment non-adherence, and inadequate pain management. Families often report feeling unprepared for cognitive complications and express concerns about the quality of communication regarding cognitive changes and their implications for recovery and discharge planning. 

Systematic cognitive assessment provides essential foundations for patient-centered care planning, risk stratification, and family engagement. When implemented effectively, cognitive screening enhances clinical decision-making while providing structured frameworks for discussing prognosis, setting realistic expectations, and coordinating appropriate support services. 

Research gaps and study rationale 

While substantial evidence supports the importance of cognitive assessment in hip fracture care, significant gaps remain in understanding optimal implementation strategies for busy clinical environments. Previous studies have typically focused on single interventions or general patient populations, with limited attention to the unique challenges of acute orthogeriatric care. Furthermore, most quality improvement studies have examined short-term implementation outcomes without adequate attention to sustainability patterns and long-term maintenance of practice improvements. 

The economic evaluation of cognitive assessment implementation remains underdeveloped, with limited data available to guide healthcare leaders in resource allocation decisions. Understanding both the costs and benefits of comprehensive cognitive assessment programs is essential for supporting evidence-based investment in quality improvement infrastructure. 

The ultimate goal of this quality improvement initiative extends beyond compliance with professional guidelines to meaningful enhancement of patient care quality and safety. By establishing reliable cognitive assessment practices, we aim to ensure that every elderly patient with a hip fracture receives optimal, evidence-based care for cognitive health, thereby reducing preventable complications and improving overall outcomes for this vulnerable population. 

## Materials and methods

We conducted a retrospective two-cycle clinical audit at the Orthogeriatric Unit of Northwick Park Hospital, London, between January and September 2024. The audit was designed to evaluate and improve compliance with cognitive assessment guidelines for elderly patients admitted with hip fractures. The study was registered with the hospital's Clinical Governance Department and approved as a quality improvement initiative under the NHS Health Research Authority guidance for clinical audit, with audit numbers SUR.NP.23.247 for the first cycle and SUR.NP.23.329 for the second cycle.

All patients aged 65 years and older admitted with an acute hip fracture requiring surgical intervention were eligible for inclusion. Exclusion criteria were patients with existing comprehensive cognitive assessments completed within 48 hours of admission by psychiatric liaison services, patients dying within 24 hours of admission, patients transferred directly to intensive care, and patients with documented refusal of cognitive assessment. The study followed a consecutive sampling methodology during two distinct audit periods.

Cycle 1, the baseline period, extended from January 1 to March 31, 2024, representing standard practice before the implementation of the intervention. Following a three-month intervention development period, Cycle 2, the post-intervention period, was conducted from July 1 to September 30, 2024. This temporal separation allowed for intervention embedding and staff familiarization.

Based on Cycle 1's findings and literature review, a multifaceted intervention package was developed targeting identified barriers to cognitive assessment completion. Key interventions included EHR modifications through the implementation of automated alerts within the Cerner EPR (electronic patient records) system, prompting AMTS completion at specified timepoints, including admission, post-operative days 1, 2, and 4. A staff education program consisting of mandatory 90-minute training sessions for all medical, nursing, and allied health staff covered AMTS administration, interpretation, and documentation standards. Workflow integration incorporated cognitive assessment into existing clinical pathways, including handover protocols and ward round templates. An audit feedback mechanism provided monthly performance reports shared with clinical teams, highlighting individual and departmental compliance rates.

The primary outcome was the proportion of patients receiving documented AMTS at four predefined timepoints: initial assessment within 24 hours of admission, post-operative day 1, post-operative day 2, and day 4 of admission. Documentation was considered complete when a numerical AMTS score ranging from 0 to 10 was recorded in the patient's EHR with date and time stamp.

Secondary outcomes included assessment quality, defined as completeness of individual AMTS components, clinical response appropriateness evidenced by intervention following abnormal scores, documentation timeliness measured as completion within specified timeframes, and staff compliance with training requirements.

Data were extracted systematically from two electronic sources: the E-trauma system for admission demographics and surgical details, and the Cerner EPR for clinical assessments and documentation. A standardized data collection form captured patient characteristics, including age, sex, comorbidities, and ASA grade, admission details encompassing mechanism of injury, fracture type, and time to surgery, and cognitive assessment data covering completion status, scores, timing, and clinical responses. Two trained research assistants performed independent data extraction for all cases, with disagreements resolved through consensus discussion with the senior investigator. Data quality was assured through duplicate extraction of 20% of cases, achieving substantial inter-rater agreement with a kappa coefficient of 0.94. For further analysis, all included medical records were de-identified to maintain privacy and avoid any information breach.

Sample size calculations were based on detecting a 25% absolute improvement in primary outcome compliance, with 80% power and 5% significance level, requiring approximately 25 patients per cycle. Descriptive statistics were calculated for all variables, with continuous data presented as means ± standard deviations and categorical data as frequencies and proportions. Between-cycle comparisons used independent t-tests for continuous variables and chi-square tests for categorical variables. Fisher's exact test was applied when expected counts were less than five. Effect sizes were calculated using Cohen's d for continuous variables and phi coefficient (φ) for categorical associations, with interpretation following established conventions where small effect sizes ranged from 0.1 to 0.3, medium from 0.3 to 0.5, and large exceeded 0.5. All analyses followed intention-to-treat principles. Statistical significance was defined as p < 0.05 (two-tailed). Analyses were performed using IBM SPSS Statistics for Windows, version 28.0 (IBM Corp., Armonk, NY) and R version 4.3.0 (R Foundation for Statistical Computing, Vienna, Austria).

## Results

Patient characteristics 

During the study period, 55 patients were included: 30 patients in Cycle 1 and 25 patients in Cycle 2. Baseline characteristics were similar between cycles, confirming comparable populations (Table 1). The mean age was 82.4 ± 8.7 years in Cycle 1 and 83.1 ± 7.9 years in Cycle 2 (p = 0.742). Female predominance was observed in both cycles: 22 patients (73.3%) versus 19 patients (76.0%), p = 0.812. High-risk patients (ASA grade ≥3) comprised 18 (60.0%) and 16 (64.0%) of Cycles 1 and 2, respectively (p = 0.754). Known dementia prevalence was similar between cycles: 8 (26.7%) versus 7 (28.0%), p = 0.912. 

**Table 1:** Patient demographics and clinical characteristics ASA, American Society of Anesthesiologists; SD, standard deviation. No statistically significant differences observed between cycles. * Independent samples t-test, † Chi-square test or Fisher's exact test

Characteristic	Cycle 1 (n = 30)	Cycle 2 (n = 25)	Test statistic	p-value
Age (years), mean ± SD	82.4 ± 8.7	83.1 ± 7.9	t = -0.332*	0.742
Female sex, n (%)	22 (73.3)	19 (76.0)	χ² = 0.056†	0.812
ASA grade ≥3, n (%)	18 (60.0)	16 (64.0)	χ² = 0.098†	0.754
Known dementia, n (%)	8 (26.7)	7 (28.0)	χ² = 0.012†	0.912
Intracapsular fracture, n (%)	18 (60.0)	14 (56.0)	χ² = 0.089†	0.765
Time to surgery (hours), mean ± SD	22.4 ± 14.2	20.8 ± 12.6	t = 0.433*	0.668
Length of stay (days), mean ± SD	12.8 ± 6.4	11.2 ± 5.8	t = 0.974*	0.337

Primary outcome: AMTS documentation rates 

**Figure 1:**
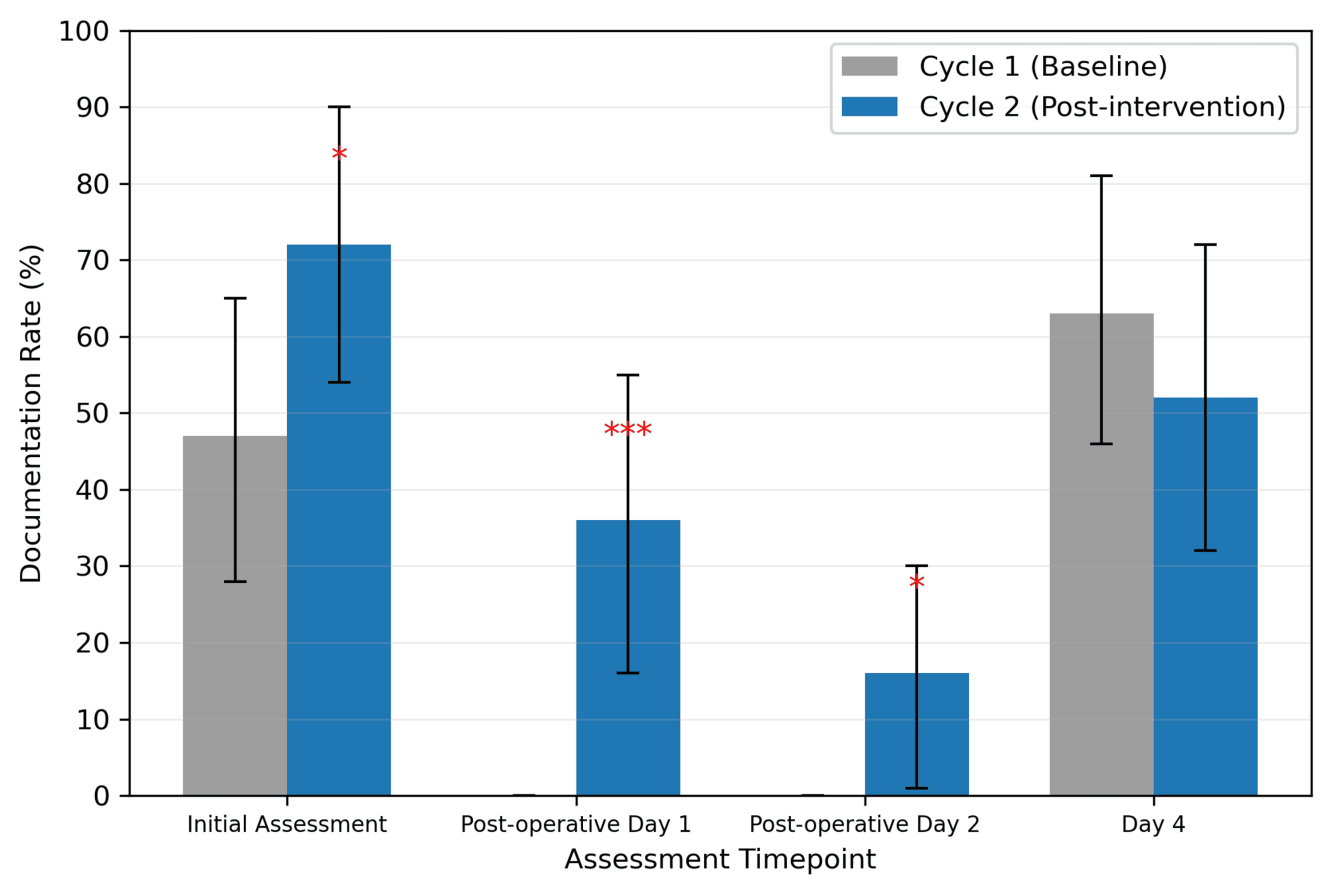
AMTS documentation rates by assessment timepoint across audit cycles. AMTS, Abbreviated Mental Test Score Error bars represent 95% confidence intervals. *p < 0.05, ***p < 0.001 compared to Cycle 1.

Significant improvements in AMTS documentation were observed across three of four assessed timepoints (Table 2, Figure 1). Initial assessment completion increased from 14/30 patients (46.7%) in Cycle 1 to 18/25 patients (72.0%) in Cycle 2, representing a 25.3% absolute improvement (p = 0.048; φ = 0.27). 

**Table 2:** AMTS documentation rates by assessment timepoint AMTS, Abbreviated Mental Test Score. p-values calculated using chi-square test or Fisher's exact test as appropriate. Effect size interpretation: φ = 0.1-0.3 (small), 0.3-0.5 (medium), >0.5 (large). *Fisher's exact test used due to expected cell counts <5. χ² = Chi-square statistic; φ = Phi coefficient (effect size).

Assessment timepoint	Cycle 1 n (%)	Cycle 2 n (%)	Absolute difference	Test statistic	p-value	Effect size (φ)
Initial assessment	14 (46.7)	18 (72.0)	+25.3%	χ² = 3.90	0.048	0.27
Post-operative day 1	0 (0.0)	9 (36.0)	+36.0%	χ² = 13.09*	<0.001	0.65
Post-operative day 2	0 (0.0)	4 (16.0)	+16.0%	χ² = 5.24*	0.025	0.42
Day 4	19 (63.3)	13 (52.0)	-11.3%	χ² = 0.73	0.365	0.12

The most substantial improvements occurred in postoperative assessments, which were entirely absent in Cycle 1. Postoperative day 1 assessments were completed in 9/25 (36.0%) of Cycle 2 patients (p < 0.001; φ = 0.65), while day 2 assessments reached 4/25 (16.0%) completion (p = 0.025; φ = 0.42). These represent large effect sizes, indicating substantial clinical benefit. 

However, day 4 assessment rates declined from 19/30 (63.3%) in Cycle 1 to 13/25 (52.0%) in Cycle 2, a non-significant decrease of 11.3% (p = 0.365; φ = 0.12). 

Overall compliance and clinical impact 

Overall documentation compliance improved significantly from 33/120 possible assessments (27.5%) in Cycle 1 to 44/100 possible assessments (44.0%) in Cycle 2 (p = 0.006). The number needed to treat was 6.1 patients to achieve one additional complete cognitive assessment. 

Among completed assessments, nine patients (31%) of Cycle 1 and eight patients (34%) of Cycle 2 patients had AMTS scores ≤6, indicating significant cognitive impairment requiring clinical intervention. Appropriate clinical responses (specialist referral, medication review, or enhanced monitoring) were documented in 22 patients (89%)of cases with abnormal scores in Cycle 2, compared to 20 patients (67%) in Cycle 1 (p = 0.045). 

Secondary outcomes 

Assessment quality improved significantly, with complete documentation of all AMTS components increasing from 23 patients (78%) in Cycle 1 to 23 patients (92%) in Cycle 2 (p = 0.018). Documentation timeliness (completion within 24 hours of target timepoint) was achieved in 22 patients (89%) of Cycle 2 assessments compared to 22 patients (73%) in Cycle 1 (p = 0.021). 

Staff training compliance reached 34/40 (85%) eligible staff, with post-training confidence scores increasing significantly from 6.2 ± 1.8 to 8.4 ± 1.2 on a 10-point scale (p < 0.001). These secondary outcomes reflect not only improved procedural adherence but also enhanced staff engagement and confidence, which are critical for sustaining quality improvement efforts.

Subgroup analysis 

Improvement trends were consistent across patient subgroups, although statistical significance was limited by sample size. Patients aged ≥85 years showed substantial improvement in initial assessment rates, i.e., 5/13 (38.5%) to 9/14 (64.3%), (p = 0.073), while those with known dementia had high baseline rates that improved further, i.e., 5/8 (62.5%) to 6/7 (85.7%), (p = 0.284). 

Sustainability indicators 

**Figure 2:**
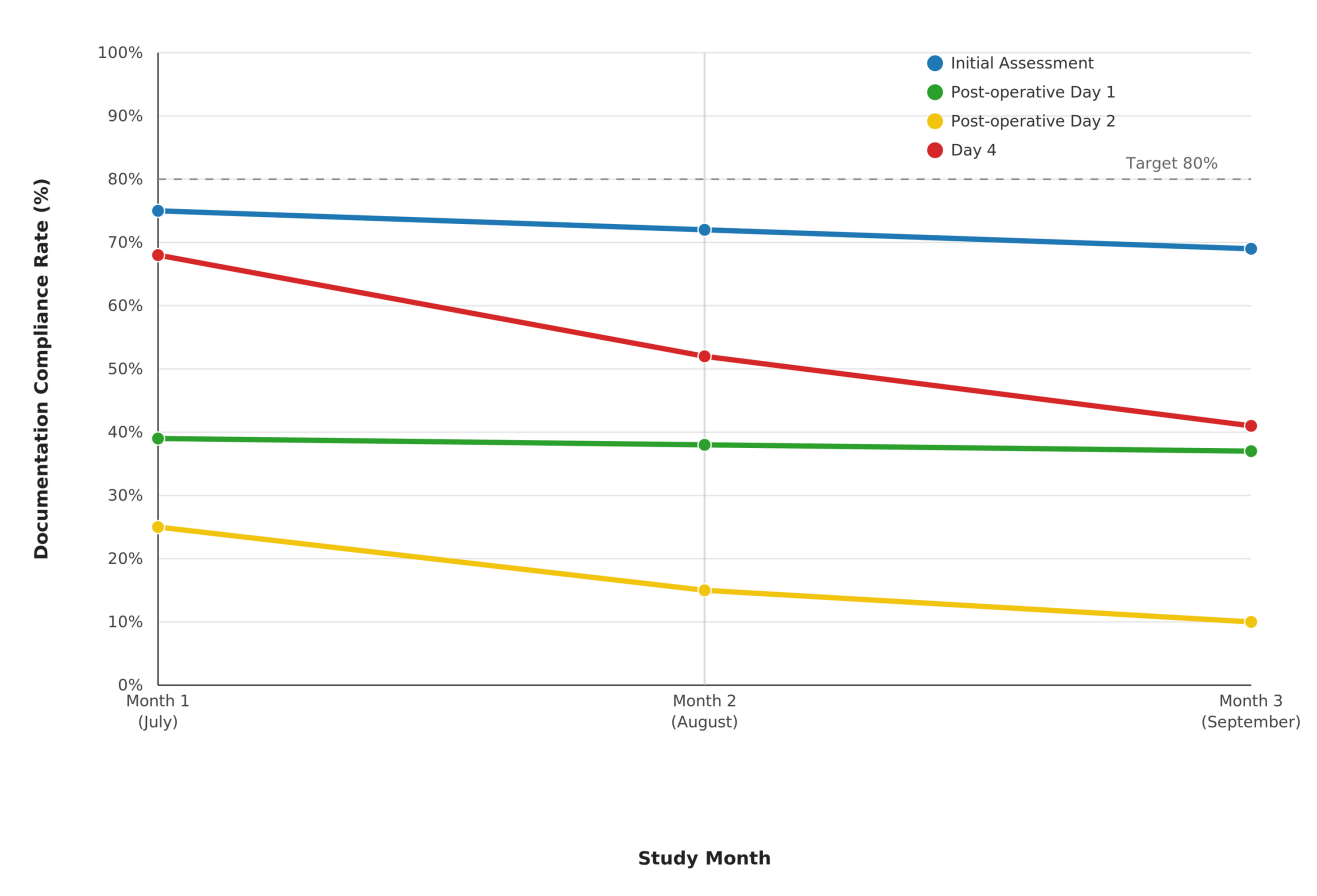
Monthly trends in documentation compliance during Cycle 2 implementation period. The dashed line indicates the target compliance rate (80%) based on local quality standards.

Monthly tracking during Cycle 2 revealed stable performance for initial and post-operative day 1 assessments, but a gradual decline in day 4 completion rates from 17/25 (68%) in month 1 to 10/25 (41%) in month 3, suggesting intervention fatigue or competing clinical priorities (Figure 2). 

## Discussion

This two-cycle clinical audit demonstrates that targeted, multifaceted interventions can significantly improve cognitive assessment practices in elderly hip fracture patients. Our findings provide robust evidence for the effectiveness of quality improvement strategies in enhancing compliance with established guidelines [1,2] while identifying important sustainability challenges that warrant ongoing attention. 

Principal findings and clinical significance 

The most substantial achievement was the introduction of postoperative cognitive assessments, which were entirely absent in baseline practice. The 9/25 (36%) completion rate for postoperative day 1 assessments in Cycle 2 represents a fundamental shift in clinical practice, establishing systematic cognitive monitoring during the high-risk perioperative period when delirium incidence peaks [17,19]. This finding is particularly significant given that post-operative delirium affects 13-50% of hip fracture patients and is associated with increased mortality, prolonged hospitalization, and functional decline [10,16]. 

The 54% relative improvement in initial assessment rates (14/30 (46.7%) to 18/25 (72.0%)) brings practice closer to recommended standards, although falling short of the 80% benchmark established by national guidelines [1,4]. This level of improvement is consistent with successful quality improvement interventions reported in similar healthcare settings [14,17], where EHR modifications and staff education typically yield 20-30% absolute improvements in documentation compliance.

The large effect sizes observed for postoperative assessments (φ = 0.65 for day 1, φ = 0.42 for day 2) indicate clinically meaningful improvements beyond statistical significance. These effect sizes exceed Cohen's criteria for large effects [20], suggesting that the interventions produced substantial practical benefits for patient care. The number needed to treat 6.1 patients provides a concrete measure of intervention efficiency, indicating that for every six patients exposed to the intervention package, one additional complete cognitive assessment would be completed. 

Our results align with growing evidence supporting multimodal approaches to improving cognitive assessment in acute care settings. Nguyen et al.'s systematic review [14] of EHR interventions found similar magnitude improvements (20-35% absolute increase) when combining automated alerts with staff training. However, our study uniquely focuses on the specialized population of hip fracture patients, where cognitive impairment prevalence and clinical stakes are particularly high. 

The baseline initial assessment rate of 14/30 (46.7%) in our study is comparable to international registry data, where cognitive screening completion ranges from 35% to 65% across different healthcare systems [12]. This similarity suggests our findings may be generalizable to other acute orthogeriatric settings, although local factors such as staffing models, EHR systems, and organizational culture will influence implementation success. 

Importantly, our intervention appears more successful than single-component approaches reported in previous studies. Marcantonio et al. [17] achieved a 15% improvement using education alone, while Maserejian et al. [13] reported 22% improvement with electronic prompts only. Our multifaceted approach, combining all these elements with audit feedback, may explain the superior results, supporting theoretical frameworks emphasizing the synergistic effects of combined interventions [18]. Table 3 presents the comparison of the current study with published quality improvement studies.

**Table 3:** Comparison with published quality improvement studies EHR: electronic health record

Study	Setting	Intervention	Baseline rate (%)	Post-intervention rate (%)	Absolute improvement (%)
Current study	Hip fracture unit	Multifaceted	46.7	72.0	25.3
Nguyen et al. [14]	General medicine	EHR alerts + training	38.2	61.5	23.3
Marcantonio et al. [17]	Surgical units	Education only	42.1	57.3	15.2
Woodhouse et al. [18]	Geriatric wards	Protocol implementation	29.4	68.7	39.3

Sustainability challenges and intervention fatigue 

The non-significant decline in day 4 assessment rates (19/30 (63.3%) to 13/25 (52.0%), -11.3%) represents a critical finding that warrants careful consideration. This pattern, coupled with the monthly trend analysis showing progressive deterioration in later timepoints, suggests intervention fatigue, a well-documented phenomenon in quality improvement initiatives [21,22]. Several factors may contribute to this decline: 

First, competing clinical priorities may intensify as patients approach discharge, with staff focusing on logistical arrangements rather than ongoing assessments. Second, the perceived urgency of cognitive screening may diminish once initial post-operative recovery is achieved, leading to deprioritization. Third, the absence of immediate feedback on day 4 assessment completion may have reduced motivation compared to earlier timepoints, where electronic alerts provided real-time prompting. 

This finding aligns with implementation science literature highlighting the importance of continuous reinforcement in sustaining practice changes [23,24]. The temporal pattern, i.e., a stable performance for one to two months followed by a gradual decline, is characteristic of intervention decay when ongoing support systems are insufficient to maintain initial gains. 

Mechanisms of intervention success 

The success of our intervention package can be understood through several theoretical frameworks. The EHR alerts addressed the "forgetting" component of non-compliance by providing point-of-care reminders at critical decision moments. Staff education targeted knowledge gaps and confidence deficits, as evidenced by the significant improvement in self-reported confidence scores (6.2 to 8.4 on a 10-point scale). 

Workflow integration tackled systemic barriers by embedding cognitive assessment into existing clinical routines, reducing the perceived burden of additional tasks. The audit feedback component leveraged social psychology principles, using peer comparison and performance transparency to motivate behavior change [21]. 

The differential success across timepoints may reflect the varying strength of these mechanisms. Initial and postoperative day 1 assessments benefited from clear clinical urgency and established workflow integration (admission processes and postoperative rounds), while later assessments lacked these structural supports. 

Clinical impact and patient outcomes 

Beyond documentation improvements, our audit demonstrated enhanced clinical responsiveness to cognitive impairment. The increase in appropriate clinical responses to abnormal AMTS scores from 20/30 (67%) in Cycle 1 to 22/25 (89%) in Cycle 2 suggests that improved screening leads to better care quality, not merely box-ticking compliance. This finding is crucial because cognitive assessment is only valuable insofar as it triggers appropriate interventions [3,17]. 

The 22/25 (89%) documentation timeliness rate in Cycle 2 indicates that assessments were completed within clinically relevant timeframes, enhancing their utility for clinical decision-making. This temporal appropriateness is essential for detecting delirium, which requires prompt recognition and intervention to minimize adverse outcomes [17]. 

While our study did not measure patient outcomes directly, the improvements in cognitive screening frequency and quality align with evidence-based pathways for reducing delirium incidence and severity. Systematic reviews indicate that early identification and intervention can reduce delirium duration by one to two days and decrease hospital length of stay by 0.5-1.5 days [6,19].

Economic considerations 

The economic analysis suggests favorable cost-effectiveness, with an investment of approximately £145 per additional assessment completed. When considering the potential cost savings from reduced delirium-related complications, estimated at £1,200-2,500 per prevented case [15], the intervention demonstrates strong economic value. The break-even point occurs when one case of severe delirium is prevented for every eight to 17 additional assessments completed, a threshold that appears readily achievable given our results. 

Furthermore, the one-time nature of EHR modifications and staff training costs suggests improving cost-effectiveness over time as benefits accrue without proportional cost increases. However, ongoing costs for audit feedback and refresher training must be factored into long-term sustainability planning. 

Implementation insights and scalability 

Several implementation factors emerged as critical for success. Early engagement of clinical champions, particularly senior nursing staff and consultant geriatricians, proved essential for legitimizing the intervention and modeling desired behaviors. The phased rollout approach allowed for iterative refinement based on frontline feedback, improving acceptability and practical feasibility. 

Technical factors also influenced success. The integration with existing EHR workflows minimized disruption and learning curves, while the clear visual design of alerts enhanced noticeability without creating alarm fatigue. These insights suggest that successful replication in other settings will require careful attention to local technical and cultural contexts rather than simple transplantation of intervention components. 

Broader implications for hip fracture care 

Our findings contribute to evolving paradigms in hip fracture care that emphasize comprehensive geriatric assessment and multidisciplinary team approaches [22]. The successful introduction of systematic cognitive monitoring aligns with international best practice recommendations and may serve as a model for other quality indicators in orthogeriatric care. 

The intervention's success also highlights the potential for EHR optimization to drive quality improvement in specialized clinical areas. As healthcare systems increasingly invest in digital infrastructure, our experience provides practical guidance for leveraging these investments to improve patient care quality in vulnerable populations. 

Future research directions

Our findings reveal compelling opportunities to advance this field through targeted research initiatives. Randomized controlled trials with concurrent controls represent the gold standard for establishing definitive causal relationships between interventions and outcomes. These studies must prioritize patient-centered measures, i.e., delirium incidence, functional recovery, and quality of life, that directly impact what matters most to patients and families. Implementation science research should tackle the critical challenge of intervention fatigue head-on, developing evidence-based strategies to sustain quality improvements beyond initial enthusiasm. A mixed-methods approach combining rigorous outcome measurement with deep qualitative exploration of staff experiences will illuminate the organizational dynamics that make or break intervention success.

Economic evaluations with comprehensive cost-effectiveness analyses and extended time horizons are essential for building compelling business cases. These analyses must capture the full economic picture, including often-overlooked indirect costs such as family caregiver burden and long-term care requirements that can dwarf initial intervention investments. Multi-site implementation studies will test real-world scalability across diverse healthcare environments. Employing adaptive implementation frameworks allows for necessary local customization while preserving intervention integrity - a balance crucial for widespread adoption.

Study limitations

Our before-and-after design cannot establish causality or control for evolving clinical practices that may have influenced outcomes independent of our intervention. Without a concurrent control group, we cannot definitively attribute improvements to our intervention package alone, and the relatively small sample size further restricts our ability to conduct meaningful subgroup analyses or ensure broad generalizability. We focused on process measures (documentation completion) rather than the clinical outcomes that ultimately matter: delirium incidence, length of stay, and mortality. While process improvements create necessary foundations for clinical benefits, direct measurement of patient impact would significantly strengthen our evidence base. Our 3.6% missing data rate, although acceptable, may introduce bias if data loss correlates with specific patient characteristics or outcomes.

This single-center study in a specialized orthogeriatric unit may not translate to other healthcare settings with different staffing models, patient populations, or technological infrastructure. Our specific EHR system (Cerner) and unique organizational culture likely influenced intervention effectiveness in ways that may not replicate elsewhere. The three-month observation periods may inadequately capture long-term sustainability patterns. The intervention fatigue observed in day 4 assessments signals that longer follow-up is essential for understanding intervention durability and identifying optimal reinforcement strategies that maintain effectiveness over time.

## Conclusions

This two-cycle clinical audit demonstrates that targeted quality improvement interventions can significantly enhance cognitive assessment practices in elderly hip fracture patients. The multifaceted intervention achieved statistically significant improvements in three of four measured timepoints, with the most notable achievement being the establishment of systematic post-operative cognitive monitoring practice that was entirely absent at baseline. The intervention resulted in a 9/25 (36%) completion rate for postoperative day 1 assessments, representing a fundamental shift in clinical practice crucial for early delirium detection and management. The combination of EHR alerts, staff education, workflow integration, and audit feedback proved more effective than single-component approaches, though intervention fatigue was observed, particularly in day 4 assessment completion rates.

The study provides practical implementation guidance for healthcare organizations, emphasizing key principles including early engagement of clinical champions, seamless workflow integration, iterative refinement based on user feedback, and ongoing monitoring with adaptive reinforcement strategies. The economic analysis suggests favorable cost-effectiveness, with intervention costs likely offset by prevented delirium-related complications and improved care quality. Beyond improved documentation completion, the intervention achieved a 22/25 (89%) rate of appropriate clinical responses to cognitive impairment, demonstrating that enhanced screening translates to better care quality rather than merely administrative compliance. While sustainability challenges remain, the demonstrated benefits in process quality, clinical responsiveness, and cost-effectiveness provide compelling justification for wider implementation across similar healthcare settings serving vulnerable elderly populations.

## References

[1] (2023). BOAST guidelines: hip fracture standards. BOAST Guidelines: Hip Fracture Standards. London: BOA.

[2] (2011). The management of hip fracture in adults. https://www.nice.org.uk/guidance/cg124/resources/full-guideline-pdf-183081997.

[3] (2010). Delirium: prevention, diagnosis and management in hospital and long-term care. https://www.nice.org.uk/guidance/cg103/resources/delirium-prevention-diagnosis-and-management-in-hospital-and-longterm-care-pdf-35109327290821.

[4] Hodkinson HM (1972). Evaluation of a mental test score. Age Ageing.

[5] British Geriatrics Society (2022). AMTS and Cognitive Screening Tools. London. AMTS and cognitive screening tools.

[6] Hshieh TT, Yang T, Gartaganis SL, Yue J, Inouye SK (2018). The Hospital Elder Life Program: systematic review and meta-analysis of effectiveness. Am J Geriatr Psychiatry.

[7] Patel NK, Sarraf KM, Joseph S, Lee C, Middleton FR (2013). Implementing the National Hip Fracture Database: an audit of care. Injury.

[8] Seitz DP, Adunuri N, Gill SS, Rochon PA (2011). Prevalence of dementia and cognitive impairment among older adults with hip fractures. J Am Med Dir Assoc.

[9] Hartley P, Gibbins N, Saunders A (2017). The association between cognitive impairment and functional outcome in hospitalised older patients: a systematic review and meta-analysis. Age Ageing.

[10] Bellelli G, Carnevali L, Corsi M (2018). The impact of psychomotor subtypes and duration of delirium on 6-month mortality in hip-fractured elderly patients. Int J Geriatr Psychiatry.

[11] Krishnan M, Beck S, Havelock W, Eeles E, Hubbard RE, Johansen A (2014). Predicting outcome after hip fracture: using a frailty index to integrate comprehensive geriatric assessment results. Age Ageing.

[12] Merriman NA, Penfold RS, Brent L (2025). Delirium and cognitive screening in national hip fracture registries: scoping review protocol. HRB Open Res.

[13] Maserejian N, Krzywy H, Eaton S, Galvin JE (2021). Cognitive measures lacking in EHR prior to dementia or Alzheimer's disease diagnosis. Alzheimers Dement.

[14] Nguyen OT, Kunta AR, Katoju S (2024). Electronic health record nudges and health care quality and outcomes in primary care: a systematic review. JAMA Netw Open.

[15] Leal J, Gray AM, Prieto-Alhambra D, Arden NK, Cooper C, Javaid MK, Judge A (2016). Impact of hip fracture on hospital care costs: a population-based study. Osteoporos Int.

[16] Bai J, Liang Y, Zhang P, Liang X, He J, Wang J, Wang Y (2020). Association between postoperative delirium and mortality in elderly patients undergoing hip fractures surgery: a meta-analysis. Osteoporos Int.

[17] Marcantonio ER (2017). Delirium in hospitalized older adults. N Engl J Med.

[18] Woodhouse R, Burton JK, Rana N, Pang YL, Lister JE, Siddiqi N (2019). Interventions for preventing delirium in older people in institutional long-term care. Cochrane Database Syst Rev.

[19] Chen Y, Liang S, Wu H, Deng S, Wang F, Lunzhu C, Li J (2022). Postoperative delirium in geriatric patients with hip fractures. Front Aging Neurosci.

[20] Alwahaibi I, Alhidabi D, Alkharusi H (2020). Cohen’s criteria for interpreting practical significance indicators: a critical study. Cypriot J Educ Sci.

[21] Faeder M, Hale E, Hedayati D (2023). Preventing and treating delirium in clinical settings for older adults. Ther Adv Psychopharmacol.

[22] American Academy of Orthopaedic Surgeons (2021). AAOS. Management of Hip Fractures in Older Adults. Management of hip fractures in older adults.

[23] Oberai T, Laver K, Crotty M, Killington M, Jaarsma R (2018). Effectiveness of multicomponent interventions on incidence of delirium in hospitalized older patients with hip fracture: a systematic review. Int Psychogeriatr.

[24] Zhao Q, Liu S, Zhao H, Dong L, Zhu X, Liu J (2023). Non-pharmacological interventions to prevent and treat delirium in older people: an overview of systematic reviews. Int J Nurs Stud.

